# T Regulatory Cells Are Markers of Disease Activity in Multiple Sclerosis Patients

**DOI:** 10.1371/journal.pone.0021386

**Published:** 2011-06-24

**Authors:** Dacia Dalla Libera, Diletta Di Mitri, Alessandra Bergami, Diego Centonze, Claudio Gasperini, Maria Grazia Grasso, Simona Galgani, Vittorio Martinelli, Giancarlo Comi, Carlo Avolio, Gianvito Martino, Giovanna Borsellino, Federica Sallusto, Luca Battistini, Roberto Furlan

**Affiliations:** 1 Institute of Experimental Neurology, Division of Neuroscience, San Raffaele Scientific Institute, Milan, Italy; 2 Fondazione Santa Lucia, Rome, Italy; 3 Dipartimento di Neuroscienze, Università Tor Vergata, Rome, Italy; 4 Lancisi Neurology Unit, San Camillo Hospital, Rome, Italy; 5 Department of Medical and Occupational Sciences, University of Foggia, Foggia, Italy; 6 Institute for Research in Biomedicine, Bellinzona, Switzerland; National Institutes of Health, United States of America

## Abstract

FoxP3^+^ Treg cells are believed to play a role in the occurrence of autoimmunity and in the determination of clinical recurrences. Contradictory reports are, however, available describing frequency and function of Treg cells during autoimmune diseases. We examined, by both polychromatic flow cytometry, and real-time RT-PCR, several Treg markers in peripheral blood mononuclear cells from patients with multiple sclerosis (MS), an autoimmune disease affecting the central nervous system. We found that Tregs, as defined by CD25, CD39, FoxP3, CTLA4, and GITR expression, were significantly decreased in stable MS patients as compared to healthy donors, but, surprisingly, restored to normal levels during an acute clinical attack. We conclude that Treg cells are not involved in causing clinical relapses, but rather react to inflammation in the attempt to restore homeostasis.

## Introduction

Cells with immunosuppressive functions raise particular interest in multiple sclerosis (MS) because of their potential role in pathogenesis, determination of disease course, and their prospective use in therapy [1]. CD25^+^FoxP3^+^ T regulatory cells (Tregs) have been initially characterized in experimental autoimmune encephalomyelitis (EAE), the mouse model for MS. In EAE, CD4^+^CD25^+^ cells have a clear-cut beneficial role, suppressing cytokine production by myelin-specific pathogenic T_H_1 cells, and their transfer into normal mice prior to immunization results in decreased disease severity [2]. Furthermore, anti-CD25 treatment of resistant B10S mice renders these mice susceptible to EAE [3].

In humans, controversial results have been published. Peripheral blood CD4^+^CD25^+^ Tregs, measured by flow-cytometry, have been described variably as decreased [4], or normal [5], [6] in MS patients as compared to healthy controls. Even if normal in frequency, however, Tregs in MS patients may not be normal in function, as proposed by Viglietta et al. [7], finding that has been questioned when defining Tregs with different markers [8]. The issue of Tregs functionality is especially relevant since several studies have shown that CD4^+^CD25^+^ Treg cells are increased in inflammatory sites in autoimmunity (i.e. pancreatic islets in diabetes, synovia of arthritic joints [9] and, recently, in CSF of MS patients [10] raising the question of whether these cells are functional at these locations. Established markers of Treg functionality are lacking, however polymorphisms in Tregs effector genes such as CTLA-4, GITR, FoxP3 have been linked to susceptibility to autoimmune diseases in humans, including MS [11], [12]. Misleading and contradictory data on Tregs, may be due to the ambiguous nature of the markers employed so far. In fact, one of the first proposed Treg markers, CD25 - IL2 receptor'α-chain [13], [14]- is constitutively expressed by T regs but also by activated conventional T cells, B cells and macrophages [15], [16]. Even if depletion of CD4^+^CD25^+^ T cell population leads to autoimmune disease in nude mice [17], recent findings show that up to one-third of FoxP3^+^ cells in a naïve mouse are CD25^−^ and will remain unaffected by anti-CD25 monoclonal antibody (mAb) administration) [18], [19], [20]. FoxP3 is the X-linked transcription factor of the Forkhead/winged-helix box family, more recently proposed as Treg marker [21], [22]. Its mutation leads to a fatal autoimmune lymphoproliferative disease in both humans (IPEX syndrome) and mice (scurfy mice) [23] [24], and FoxP3 was demonstrated to largely control Tregs development and functional capacity [25], [26]. Recent studies, however, show that ectopic expression of FoxP3 in mouse CD4^+^ T cells is not sufficient to generate Tregs in vitro, and transient FoxP3 expression has been demonstrated in recently activated T cells [24], [27], [28]. Treg effector molecules, which contribute to the activation and proliferation of these cells and tune their suppression ability, such as CTLA-4 and GITR, are considered as Treg functional markers. CTLA4 (Cytotoxic T-Lymphocyte Antigen 4) is a CD28-family receptor expressed mainly on CD4^+^ T cells, that inhibits T cell proliferation interfering with co-stimulatory signals [29], [30], [31], [32], [33], [34]. It is constitutively expressed on a subset of Tregs, but also on resting T cells. GITR (glucocorticoid-induced tumour necrosis factor receptor), a member of the TNF receptor superfamily, is a surface receptor molecule involved in inhibiting the suppressive activity of Tregs. It is constitutively expressed in Tregs at a higher level than in other T cells although recently activated T cells can also upregulate GITR expression in humans [17], [35].

Two novel functional Tregs markers are CD39 and CD73, ecto-nucleotidases present on the surface of lymphocytes which act in concert to hydrolyzes ATP or ADP to 5′-AMP and 5′-AMP to adenosine, a potent anti-inflammatory molecule [36], [37]. In humans they are expressed on antigen presenting cells, B cells and on a subset of human Foxp3^+^ Tregs with potent immunosuppressive properties representing activated effector/memory-like suppressor cells, namely TREM cells. Tregs from CD39-null mice show impaired suppressive properties in vitro and fail to block allograft rejection in vivo. Strikingly reduced numbers of CD39^+^ Tregs -but not of total Tregs- are found in the blood of patients suffering of RR-MS [38], and, more recently, decrease of CD39^+^ Tregs has been associated to disease progression in HIV [39].

Finally, low expression levels of the IL-7 receptor CD127, have been used to better define FoxP3^+^ Tregs, making of CD127 a useful and widely used negative Treg marker [40].

We analyzed, in unfractionated peripheral blood mononuclear cells, several different markers, namely CD25, FoxP3, CTLA-4, GITR, and CD39, in the attempt to more precisely define Tregs and to identify the pattern that best describes their modulation during MS. We compared flow cytometry, as gold standard, and real time RT-PCR, to validate the latter as a tool to be used in clinical settings, to overcome the issues related to sampling live cells for disease monitoring.

## Materials and Methods

### Patient characteristics

We enrolled 85 patients with a clinically definite MS - according to revised McDonald's criteria [41]- and a relapsing-remitting course. [42], from four different Centers in Italy, (San Raffaele Hospital, Milan; Tor Vergata, Rome; Santa Lucia Hospital, Rome; San Camillo Hospital, Rome). The Study has been approved by the Ethical Committee-HSR for the San Raffaele Scientific Institute, the Ethical Committee of the IRRCCS Fondazione Santa Lucia, the Ethical Committee Azienda Ospedaliera San Camillo-Forlanini, and the Ethical Committee of the Azienda ospedaliero - universitaria Ospedali Riuniti Foggia. All patients have signed an informed consent before blood withdrawal. Patients were between 18 and 52 years old, had a disease duration from less than one year to 29 years, a relatively mild neurological disability (EDSS<4.0) and MRI-findings typical of MS (according to Barkhof's criteria) [43] Patients with concomitant severe diseases (neoplasm, respiratory, renal, liver or cardiac failures), recurrent urinary or pulmonary infections, or pregnant women, were excluded.

Forty-two MS patients were in a stable phase of their disease - no relapse in the 6 months before sampling. The other 43 patients – namely “relapsing MS”- were enrolled while experiencing a well-defined relapse (as judged by clinical assessment by a trained neurologist), whose clinical onset was between 8 hours and 10 days. None of the patients had been treated with steroids or immunosuppressive agents in the 3 months preceding the relapse. Sixty-five gender and age-matched healthy controls, with no previous history of neurological or autoimmune disease and not concomitant infection or allergy access, were also enrolled in the study.

### Cell isolation, RNA extraction, and cDNA synthesis

PBMCs from venous blood have been separated by Ficoll density centrifugation (Lymphoprep, Axis Shield, Oslo, Norway) within 3 hours from withdrawal. Either stained for FACS analysis or washed and resuspended in TRIzol® for RNA extraction: 1 ml of TRIzol® Reagent (Sigma-Aldrich) was used per 10×10^6^ cells. RNA was extracted following the manufacturer's protocol. cDNA has been synthesized from 3 µg of RNA using the kit Ready-to-go (Amersham Biosciences) following the manufacturer's protocol, and Real-time PCR (RT-PCR) using pre-developed Taqman™ Assay Reagents (Applied Biosystems, Foster City, MA), has been used to measure the mRNA levels of the following targets: CD4, CD25, FOXP3, GITR, CTLA4, CD39 and the endogenous control GAPDH (all primers by Applied Biosystems, Foster City, MA). AU (arbitrary units) were calculated using the following formula: 2−ΔΔ C_T_ = 2−((C_T target_-C_T endogenous_)−Δ C_T_).

### Polychromatic Flow Cytometric Analysis

The following antibodies were used: hCD4, hFoxP3, hGITR, (eBioscience); hCD25, hCTLA4 (BD Biosciences); hCD39 (Miltenyi). Antibodies were used at predetermined optimal concentrations. Dead cells were excluded from analysis using LIVE/DEAD® Fixable Dead Cell Stain Kit (Invitrogen). FACS analysis was carried out on a FACSCanto, (BD Bioscience), or on a CyAn (Beckam Coulter). Data were analysed using FlowJo software (Treestar).

### Suppression assay

Human mononuclear cells were isolated by Ficoll gradient centrifugation (Pharmacia, Uppsala, Sweden). Human cells were further sorted with a MoFlo high speed cell sorter (Beckman Coulter), after staining for CD4, CD25 and CD39. Purity of sorted cells was always above 90%. Data were analyzed using FlowJo software (Treestar, Ashland, OR).

The CD4^+^CD25^high^CD39^+^ cell subset was tested in vitro for suppression in co-cultures with proliferating autologous CD4^+^CD25^neg^ responder cells. In vitro suppression assays were carried out in RPMI/10% FCS in 96-well V-bottom plates (Costar, Corning, NY). CFSE-labeled (Invitrogen, Carlsbad, CA) CD4^+^CD25^neg^ responder cells (2.0×10^4^) were incubated with titrated amounts of FACS-sorted CD4^+^CD39^+^ and 10×10^4^ irradiated (3000 rad) antigen-presenting cells (APCs) that were T-cell-depleted with α-CD3 (T3D). Stimulation was carried out with plate-bound α-CD3 (UCTH1; 10 µg/mL). After 5 days at 37°C, samples were acquired on a Cyan flow-cytometer (Beckman Coulter) and data were analyzed using FlowJo software (TreeStar, Ashland, OR) to assess cell proliferation.

### Statistical Analysis

As the datasets did not conform to a normal distribution, median percentage (± inter-quartile ranges) and nonparametric tests (Mann-Whitney) were used throughout. A probability value <0.05 was considered statistically significant. Data were analyzed with Prism (version) 5.0.

## Results

### CD4^+^CD25^+^CD39^+^T cells co-express typical Treg markers

**Figure 1:**
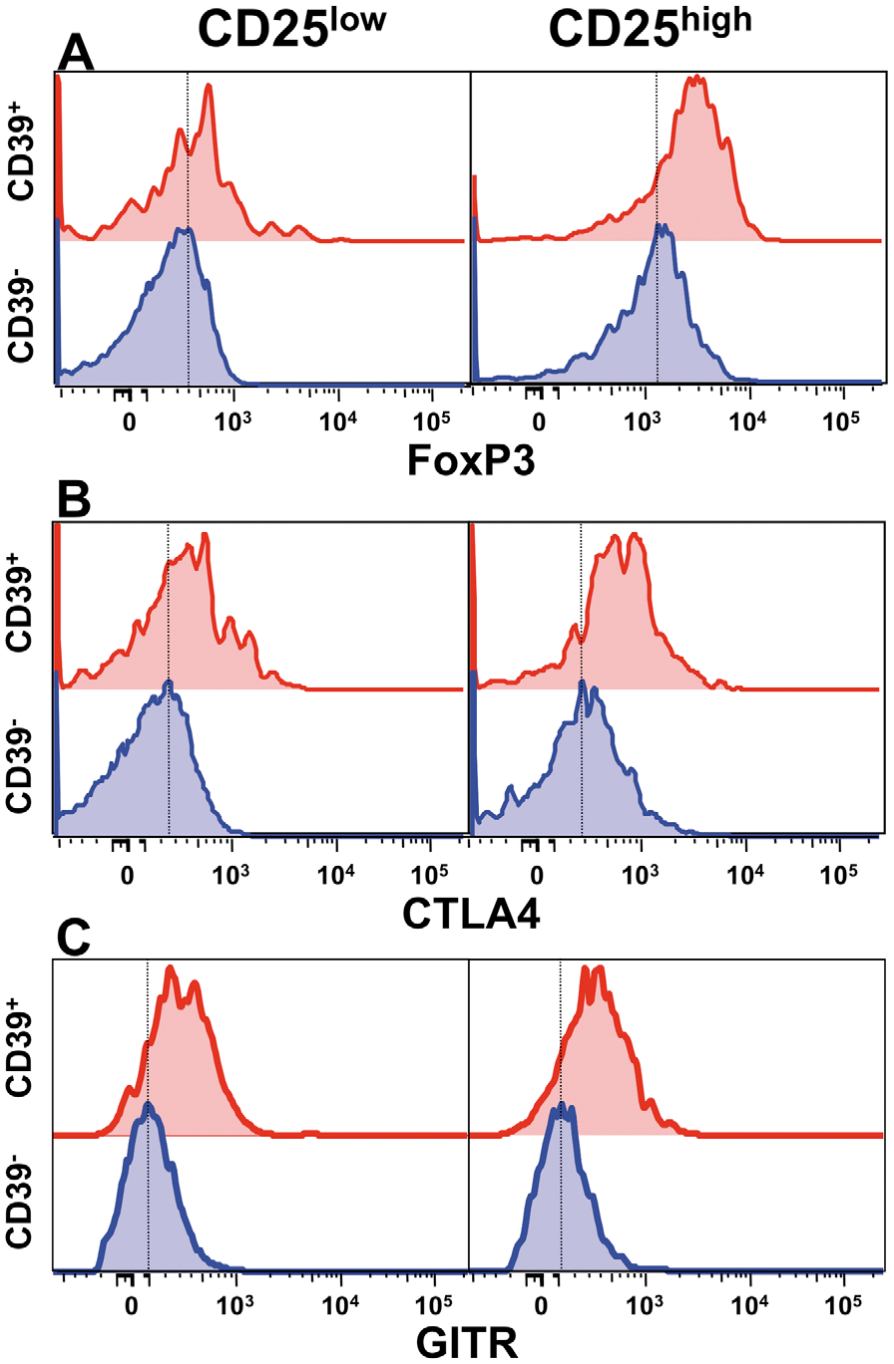
CD4^+^CD25^high^CD39^+^ T cells express increased levels of Treg markers. FoxP3 (**A**), CTLA4 (**B**) and GITR (**C**) expression, as measured by flow cytometry, are increased in CD4^+^CD39^+^, as compared to CD4^+^CD39^−^ T cells, especially in the CD25^high^ compartment.

CD4^+^CD25^high^CD39^+^ T cells are *bona fide* Treg cells, both those from healthy donors and from MS patients, displaying proliferation suppressive ability in classical *in vitro* assays [38]. In agreement with this model, when CD39^+^ T cells were compared to CD39^−^ negative T cells, we found increased levels of expression of all Treg markers tested, namely Foxp3 (Fig. 1A), CTLA4 (Fig. 1B), and GITR (Fig. 1C).

### Molecular Treg markers are elevated in MS patients experiencing a clinical relapse

**Figure 2:**
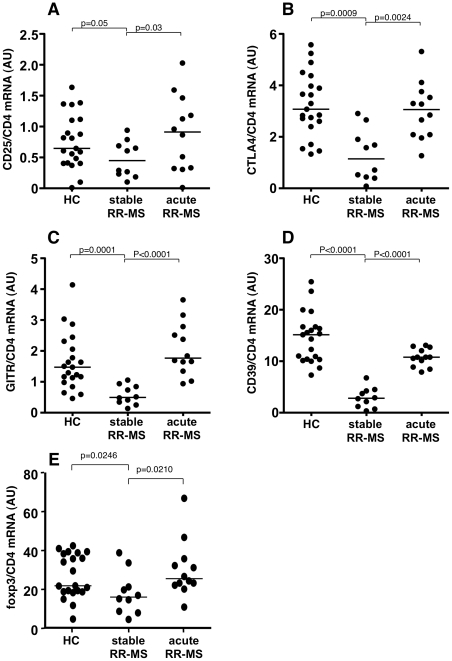
Treg markers are up-regulated in RR-MS patients experiencing clinical relapses. **A–E.** Clinically relapsing RR-MS patients displayed increased PBMC mRNA levels for CD25 (**A**), CTLA-4 (**B**), GITR (**C**), CD39 (**D**), and foxp3 (**E**). We also found significantly lower levels of Treg markers mRNA in stable RR-MS patients as compared to healthy controls (HC). Values are expressed as arbitrary units (AU). P values are indicated (Mann-Whitney).

We used the above described Treg markers to interrogate, by molecular means, PBMC samples of patients affected by relapsing-remitting MS in a stable or acute phase of the disease. We found that all markers studied were strikingly reduced in stable MS patients as compared to healthy individuals. Surprisingly, we found that CD25 (Fig. 2A), CTLA4 (Fig. 2B), GITR (Fig. 2C), and CD39 (Fig. 2D), and foxp3 (Fig. 2E) mRNA levels were significantly increased in PBMCs from MS patients experiencing a clinical relapse, as compared to patients in a stable phase of the disease.

### Treg cells frequency is increased in MS patients experiencing a clinical relapse

**Figure 3:**
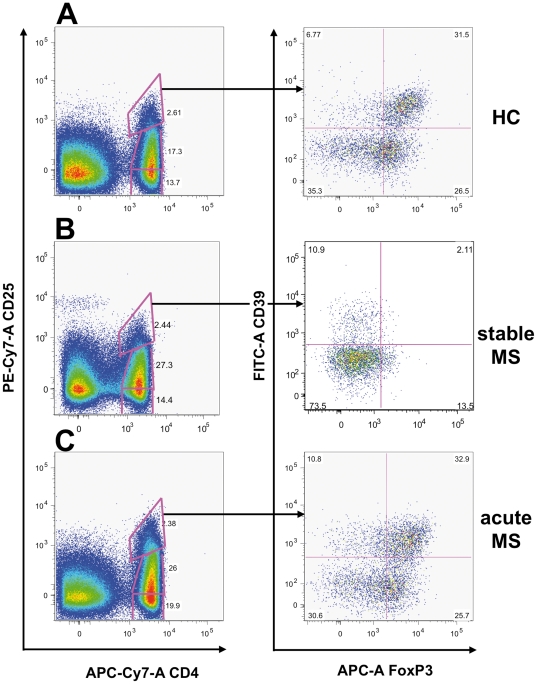
FoxP3^+^CD39^+^ Treg cells are increased during acute MS. Scatter plots from three representative subjects, one healthy donor (HC; **A**), a stable MS patients (**B**), and a patient experiencing a clinical re-exacerbation of MS (**C**) are shown, indicating that the FoxP3/CD39 double positive T cell population (**A**, right panel) in the CD25^high^ gate dramatically decreases during stable MS (**B**, right panel) and is restored during an acute attack (**C**, right panel).

**Figure 4:**
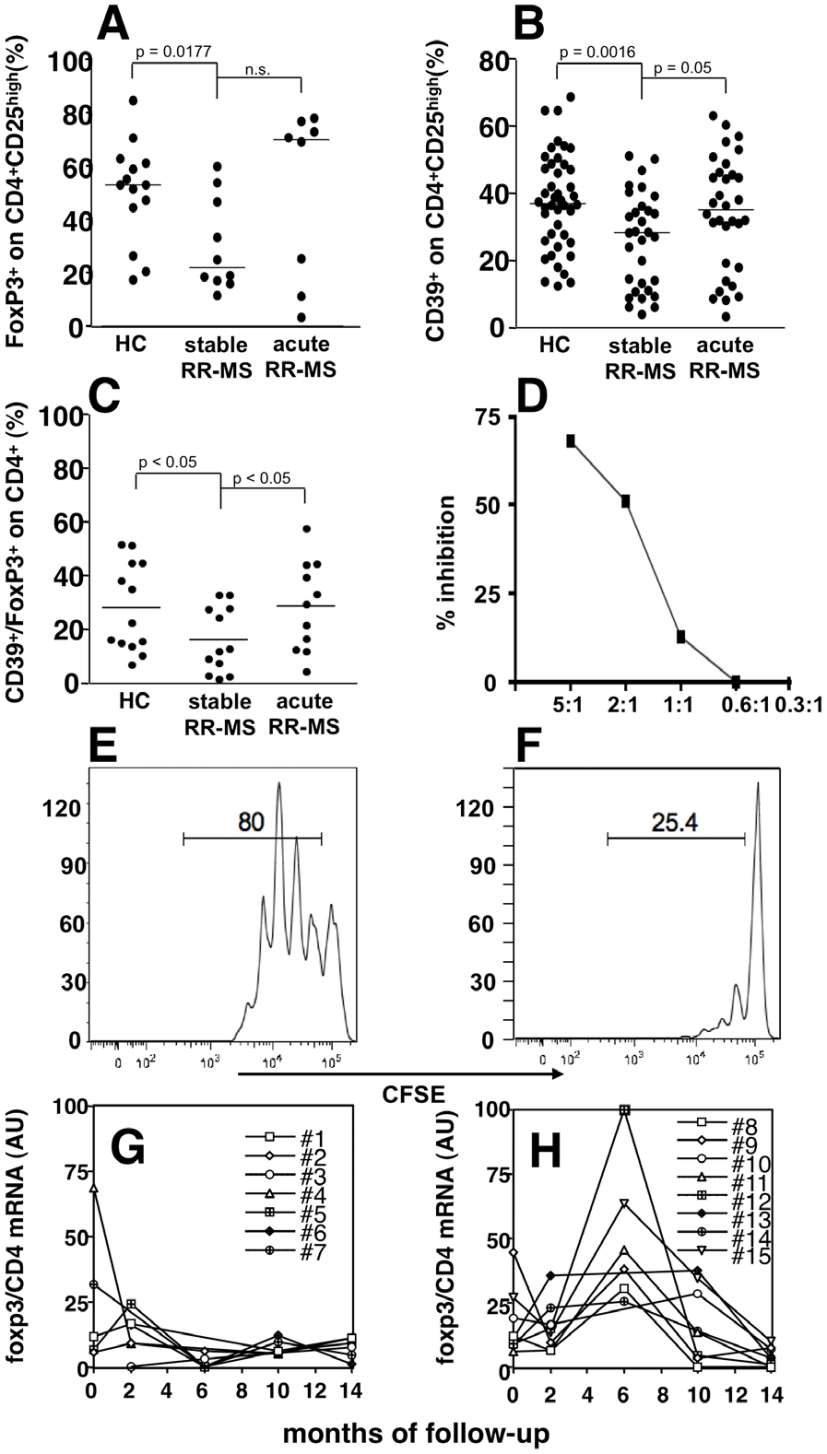
Treg cells are increased during acute MS. The percentage of FoxP3^+^ cells in the CD4^+^CD25^high^ gate is shown in (**A**) in healthy controls (HC; n = 14), stable MS (n = 10), and acute MS (n = 8). The percentage of CD39^+^ cells in the CD4^+^CD25^high^ gate is shown in (**B**) in HC (n = 44), stable MS (n = 31), and acute MS (n = 32), and, similarly, the percentage of FoxP3^+^/CD39^+^ cells in the CD4^+^CD25^high^ gate is displayed in (**C**) in HC (n = 13), stable MS (n = 12), and acute MS (n = 11). Again, while Treg cells, as defined by these markers, were significantly decreased in stable MS patients and restored during an acute attack. Lines represent median values, and P values are indicated where significant (Mann-Whitney). CD4+CD25^high^CD39^+^ regulatory T cells from an acute MS patient suppress T responder cell proliferation (E) in a dose dependent way (F), as measured by CFSE dilution assay. Plots represent CFSE-labeled T responder cell proliferation in absence and presence of regulatory T cell (5∶1) (E, F). A representative experiment among three is shown. Treatment-free RR-MS patients (n = 15) were followed longitudinally every two months for 14 months (**G–H**), and divided according to the occurrence of clinical relapses during the follow-up in stable (G), or relapsing (H) patients. PBMC *foxp3* mRNA values, normalized on CD4 and GAPDH mRNA, and expressed as arbitrary units (AU) are plotted.

To confirm that the different levels of expression of Treg markers in PBMCs of relapsing MS patients, as measured by molecular means, is associated to a corresponding modulation of the T cell population of interest, we performed polychromatic flow cytometric analysis on freshly isolated PBMCs from healthy donors and MS patients in different phases of disease activity. Since CTLA4 and GITR staining display lower discriminating sensitivity, we used CD39, FoxP3, and CD25 as previously described [38]. Indeed, stable MS patients showed a significant reduction of Treg cells as compared to healthy donors (Fig. 3A–B; Fig. 4A–C). Samples collected from patients during an acute attack, as suggested by mRNA levels, displayed restored levels of Treg cells, comparable to that observed in healthy donors (Fig. 3B–C; Fig. 4A–C). We confirmed that CD4^+^CD25^high^CD39^+^ T cells from MS patients in the acute phase of the disease are indeed suppressive, as shown in Fig. 4 D–F, thus suggesting that the T regulatory compartment is not functionally compromised in patients affected by MS.

### Longitudinally followed MS patients display increase Treg markers if experiencing a relapse

We then analyzed by RT-PCR, RNA samples from 15 untreated MS patients that had been followed longitudinally for 14 months with bi-monthly sampling, constituting the placebo arm of a clinical trial. While seven patients remained relapse-free during the follow-up period, 8 experienced on or more clinical relapses. As shown in figure 4G, patients that were clinically active during the follow-up displayed increased levels of the Treg marker Foxp3 mRNA as compared to the group of patients with stable disease, where the Foxp3 mRNA expression levels remained steady (Fig. 4H).

## Discussion

The starting point of the present work has been the observation, in archival cDNA samples, that Foxp3 mRNA levels were increased in patients experiencing a clinical relapse. Since decreased or defective Foxp3^+^ Tregs have been causally linked to relapse occurrence in several reports [4], [7], this finding appeared to be paradoxical. Recent literature has however questioned Foxp3 as Treg marker, indicating that general T cell activation implies transient Foxp3 expression [27], [28]. The increase in Foxp3 mRNA levels in relapsing MS patients can thus be attributed to increased Tregs or to increased T cell activation in broad terms. In the attempt to better distinguish between these two hypotheses, we used several different markers that have been linked to Tregs. Using this pattern of Treg-associated markers, we confirmed the observation of an apparent upregulation of the Treg compartment during disease activity in an autoimmune disease such as MS. Contradictory data are present in the literature on the number and function of Tregs in MS as compared to healthy donors, supporting the general idea that there probably is no major difference among T cells with suppressive functions that may contribute to disease development. Furthermore, peripheral blood is the only, but far from ideal, site to investigate. Considering suppressive T cells as a homeostatic mechanism aimed at controlling excessive immune activation, their secondary increase during inflammatory phases of the disease becomes reasonable and in accordance with previous observation describing them as migrating to inflammatory sites along and at the same time with other inflammatory cells [44]. We explored, at this point, the potential for these molecules to become useful biomarkers of disease activity. The fact that we found CD39, an ectonuclease thought to mediate suppressive activity of Tregs [36], [38], as one of the most reliable markers, may indicate that active, suppressive Tregs are indeed mobilized from secondary lymphoid organs as a consequence of reactivation of inflammation in the target organs, and increase in the blood when traveling to the CNS where they attempt to dampen inflammation. We propose CD39^+^ Treg as biomarkers for disease activity, but their validation is outside the scope of this work and requires larger cohorts of patients.
